# 

**Published:** 1859-03

**Authors:** 


					﻿PUBLISHED MONTHLY, AT 83 PER. ANNUM IN ADVANCE.
A T Tj A 1ST T A
ifliciii if Bii
EDITED BY
JOSEPH P. LOGAN, M. D.,
Professor of PnYsioLoor and Diseases gf Women and Children in tub Atlanta Medical
College.
and
W. F. WESTMORELAND, M. D.,
Professor op the Principles and Practice op Suroery in the Atlanta Medical College,
Pax et scientia, sed veritas sine timoro.
VoTivT MARCH, 1859.	No. VIC
ATLANTA, GEORGIA:
PRINTED BY J. 1- MILLER & CO.
(Successors to O. P. Eddy 4 Co.)
1 859.
EACH NUMBER CONTAINS SIXTY-FOUll PAGES.
				

